# Integrating Social Determinants of Health and Established Risk Factors to Predict Cardiovascular Disease Risk Among Healthy Older Adults

**DOI:** 10.1111/jgs.19440

**Published:** 2025-03-18

**Authors:** Achamyeleh Birhanu Teshale, Htet Lin Htun, Mor Vered, Alice J. Owen, Joanne Ryan, Kevan R. Polkinghorne, Monique F. Kilkenny, Andrew Tonkin, Rosanne Freak‐Poli

**Affiliations:** ^1^ School of Public Health and Preventive Medicine, Monash University Melbourne Victoria Australia; ^2^ Department of Epidemiology and Biostatistics Institute of Public Health, College of Medicine and Health Sciences, University of Gondar Gondar Ethiopia; ^3^ Department of Data Science and AI Faculty of Information Technology, Monash University Clayton Victoria Australia; ^4^ Department of Nephrology Monash Medical Centre Melbourne Victoria Australia; ^5^ Department of Medicine Monash University Melbourne Victoria Australia; ^6^ Stroke and Ageing Research, Department of Medicine School of Clinical Sciences at Monash Health, Monash University Clayton Victoria Australia; ^7^ Stroke Division The Florey Institute of Neuroscience and Mental Health, University of Melbourne Heidelberg Victoria Australia; ^8^ School of Clinical Sciences at Monash Health, Monash University Clayton Victoria Australia

**Keywords:** artificial intelligence, cardiovascular disease, deep learning, machine learning, social determinants of health

## Abstract

**Background:**

Recent evidence underscores the significant impact of social determinants of health (SDoH) on cardiovascular disease (CVD). However, available CVD risk assessment tools often neglect SDoH. This study aimed to integrate SDoH with traditional risk factors to predict CVD risk.

**Methods:**

The data was sourced from the ASPirin in Reducing Events in the Elderly (ASPREE) longitudinal study, and its sub‐study, the ASPREE Longitudinal Study of Older Persons (ALSOP). The study included 12,896 people (5884 men and 7012 women) aged 70 or older who were initially free of CVD, dementia, and independence‐limiting physical disability. The participants were followed for a median of eight years. CVD risk was predicted using state‐of‐the‐art machine learning (ML) and deep learning (DL) models: Random Survival Forest (RSF), Deepsurv, and Neural Multi‐Task Logistic Regression (NMTLR), incorporating both SDoH and traditional CVD risk factors as candidate predictors. The permutation‐based feature importance method was further utilized to assess the predictive potential of the candidate predictors.

**Results:**

Among men, the RSF model achieved relatively good performance (C‐index = 0.732, integrated brier score (IBS) = 0.071, 5‐year and 10‐year AUC = 0.657 and 0.676 respectively). For women, DeepSurv was the best‐performing model (C‐index = 0.670, IBS = 0.042, 5‐year and 10‐year AUC = 0.676 and 0.677 respectively). Regarding the contribution of the candidate predictors, for men, age, urine albumin‐to‐creatinine ratio, and smoking, along with SDoH variables, were identified as the most significant predictors of CVD. For women, SDoH variables, such as social network, living arrangement, and education, predicted CVD risk better than the traditional risk factors, with age being the exception.

**Conclusion:**

SDoH can improve the accuracy of CVD risk prediction and emerge among the main predictors for CVD. The influence of SDoH was greater for women than for men, reflecting gender‐specific impacts of SDoH.

**Summary**

- Key points:
  ○ While social determinants of health are known to influence cardiovascular disease, they have been given less attention in risk assessment tools.
  ○ In this cohort, the Random Survival Forest (RSF) model among men and the DeepSurv among women achieved good predictive performance.
  ○ Using the permutation‐based feature importance method, this study further identified social determinants of health factors as important predictors of CVD risk, especially among women.
- Why does this paper matter?
  ○ Social determinants of health have a greater role in cardiovascular disease. Incorporating them into cardiovascular disease risk assessment tools is indeed a highly commendable approach for personalized and effective cardiovascular disease interventions.

## 1 Introduction

Cardiovascular disease (CVD) is a leading cause of mortality, accounting for roughly 19.1 million deaths and constituting 32% of all global deaths [1]. Treating established CVD and early identification of high‐risk individuals could alleviate the burden [2, 3]. Therefore, the European Society of Cardiology (ESC) highlights the importance of CVD risk prediction models [4].

There have been a considerable number of multivariable CVD risk prediction models that have been published [5, 6, 7, 8]. These risk assessment tools mainly included basic demographic and traditional risk factors (e.g., age, sex, smoking, systolic blood pressure, diabetes, dyslipidemia), often neglecting social determinants of health (SDoH), which could further improve model accuracy in distinguishing high‐risk individuals [9].

SDoH are the “conditions in which people are born, grow, work, live, and age, and the wider set of forces and systems shaping the conditions of daily life” [10]. They are non‐medical factors that can have a large impact on health outcomes. The Healthy People 2030 framework [11], along with other frameworks [12, 13], identified various SDoH and categorized them into multiple domains: economic stability, education access and quality, social and community context, healthcare access and quality, and neighborhood and built environment. Recent evidence underscores the substantial influence of SDoH, which account for 30%–55% of health outcomes [14]. In the context of CVD, our recent comprehensive review demonstrates that these factors significantly affect CVD [15]. Additional studies also pointed out the effects of SDoH, such as social isolation and psychosocial factors, on CVD and its subtypes, including coronary heart disease, stroke, and heart failure [16, 17, 18].

Moreover, while risk assessment tools are recommended approaches for early detection of individuals at risk of CVD, many existing CVD risk assessment tools [19, 20, 21] often assume a linear relationship between candidate variables and CVD. However, in reality, the relationship between many risk factors, particularly SDoH variables, and CVD outcomes is non‐linear; rather, it is intertwined and complex. This highlights that erroneous methodological choices and assumptions made during prediction can result in biased estimates and decision‐making, particularly when taking SDoH into account as a risk factor. To alleviate this problem and enhance risk prediction, predictive models based on artificial intelligence (AI) are useful and have been increasingly explored [22]. For example, our recent systematic review investigating AI models for time‐to‐event outcomes used in CVD risk prediction revealed that Random survival Forest (RSF), the non‐linear Cox proportional hazards model (henceforth called DeepSurv), and Neural Multi‐Task Logistic Regression (NMTLR) models are popular and outperform the standard multivariable models such as the Cox‐proportional hazards (CoxPH) model [23]. The review also identified that SDoH variables have been overlooked in assessing the risk of CVD. Additionally, analyses that focus on gender have been given less attention in the risk prediction models that were identified. Moreover, the developed models were for younger adults, aged younger than 70 years.

Therefore, this study aims to predict gender‐specific CVD risk by integrating SDoH and traditional risk factors among older people using AI models.

## 2 Methods

### 2.1 Reporting Guideline

This study adheres to the Strengthening the Reporting of Observational Studies in Epidemiology (STROBE) and Transparent Reporting of a multivariable prediction model that uses regression or AI for Individual Prognosis Or Diagnosis (TRIPOD+AI) guidelines [24, 25].

### 2.2 Study Design and Population

This study was based on the secondary data analysis of the ASPirin in Reducing Events in the Elderly (ASPREE) trial, extended follow‐up of the ASPREE study cohort—the ASPREE eXTension (ASPREE‐XT) study, and the ASPREE Longitudinal Study of Older Persons (ALSOP). The details of these longitudinal cohorts are published elsewhere [26, 27, 28]. The participants were recruited from March 2010 through December 2014, and 19,114 older adults (aged ≥ 70 years) in Australia and the United States (including minorities aged ≥ 65 years in United States), and were free of CVD, dementia, and independence‐limiting physical disability [26]. The ALSOP, a sub‐study of ASPREE (including participants drawn from the ASPREE Australian participants), provided self‐reported data on factors that are relevant to health and well‐being in older age, such as lifestyle, socioeconomic, and environmental factors [27].

### 2.3 Variables of the Study

**FIGURE 1:**
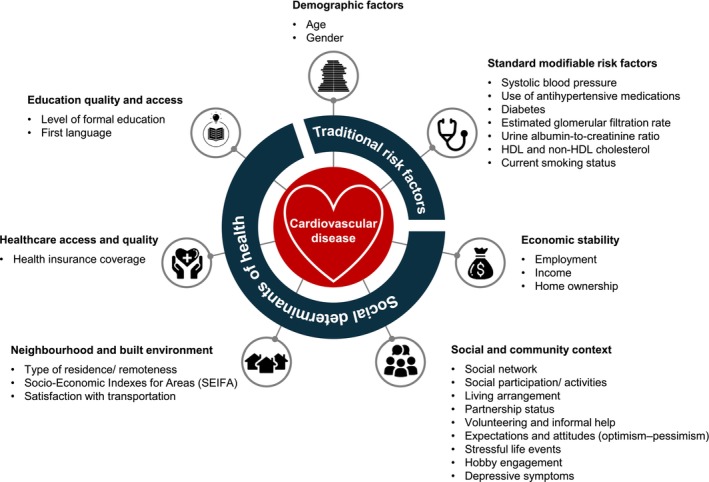
Conceptual framework: Social determinants and traditional risk factors impacting cardiovascular disease.

Our study variables included SDoH, demographic factors, and the standard modifiable CVD risk factors (Figure 1, Table S1.). Demographic factors included age, gender, and ethnicity. SDoH variables were selected based on the Healthy People 2030 [11] and the WHO conceptual SDoH [12] frameworks, as well as other published literature [29, 30, 31, 32, 33, 34, 35, 36]. These variables were then categorized into the five domains as per the Healthy People 2030 framework: Standard modifiable CVD risk factors were identified based on a *prior* risk prediction model developed in this cohort [19]. In this prior model, the traditional risk factors were identified by considering existing evidence and using a ML approach, the least absolute shrinkage and selection operator (LASSO). The study applied the standard Cox regression analyses to calculate 5‐year CVD risk and validated the model using an external validation dataset. However, this present study employed two variables to assess kidney function, estimated glomerular filtration rate (GFR) and urine albumin‐to‐creatinine ratio (ACR), in contrast to serum creatinine level used in the preceding work. This is because estimated GFR and urine ACR are more precise indicators of kidney function than serum creatinine levels alone.

### 2.4 Outcome

Our outcome of interest is the incidence of CVD. In the ASPREE study [37], CVD was an adjudicated secondary endpoint. It is a composite measure of four conditions: (1) fatal coronary heart disease, (2) non‐fatal myocardial infarction, (3) fatal or non‐fatal stroke, and (4) hospitalization due to heart failure. For this study, individuals were tracked from their time of enrollment (mentioned earlier) up to a maximum follow‐up period of approximately 12 years, up to the end of 2022.

### 2.5 Data Preprocessing

**FIGURE 2:**
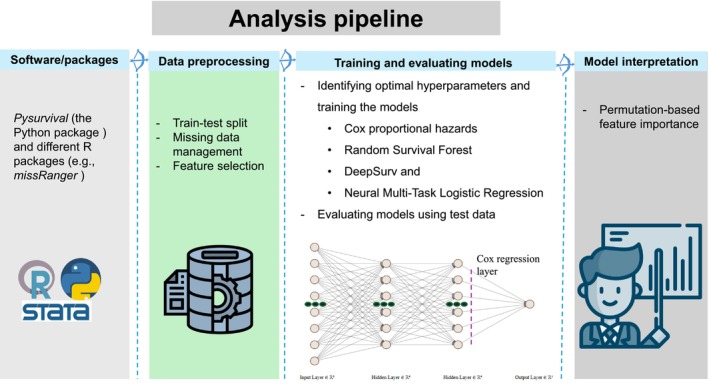
Key steps in the data analysis process.

The preprocessing included the following stages: (1) Randomly splitting the dataset into training and testing sets at a 7:3 ratio, (2) Imputing missing data using *missRanger* [38], a non‐parametric imputation based on the random forest algorithm. Table S2 presents the number and percentage of missing values, (3) Assessing multicollinearity and selecting one of two variables if their absolute correlation coefficient exceeded 0.7 [39] to reduce computational cost and prevent model overfitting, (4) Selecting relevant SDoH variables using the Elastic Net method [40], and (5) Standardizing continuous features. The details about data exploration, including model development, are depicted in Figure 2.

### 2.6 Model Development

Four models: (1) the CoxPH model, (2) RSF, (3) DeepSurv, and (4) NMTLR were considered. These ML and DL models were selected based on our recent systematic review [23]. The review revealed that RSF, DeepSurv, and NMTLR models are optimal models for CVD risk prediction [23]. RSF [41], an extension of Random Forest introduced by Breiman et al. in 2001 [42], is an ensemble tree‐based method for analyzing right‐censored survival outcomes [41]. RSF is a non‐parametric approach, which doesn't rely on assumptions like proportional hazards. The DeepSurv model is a feed‐forward model for survival analysis that combines a neural network with the partial likelihood from the CoxPH model [43]. However, unlike the conventional CoxPH model, the DeepSurv model does not rely on presumptions such as linear interactions between candidate features and the outcome. The DeepSurv model in general helps to capture complex, non‐linear feature effects while still leveraging the well‐established theory of the standard Cox model. NMTLR is a neural network model that combines the advantages of MTLR and neural networks [44]. In multi‐task learning, several logistic regression models are created for different time intervals to predict the likelihood of the event of interest occurring within each interval. Like the MTLR, the NMTLR does not rely on the proportional hazard assumption of the standard CoxPH model. However, unlike the MTLR which uses linear transformations, NMTLR incorporates a neural network, which allows for non‐linear input feature transformations. The neural network models' (the DeepSurv and NMTLR models') optimal hyperparameters, such as the network architecture, activation function, and optimizer, were determined using a random search method (repeated 100 times with 5‐fold cross‐validation) on the training dataset (Table S3).

### 2.7 Model Evaluation

The concordance index (C‐index), Integrated Brier Score (IBS), 5‐year and 10‐year area under the receiver operating characteristic (AUC), and decision curve analysis (DCA) were considered to evaluate the models. The AUC, which ranges from 0 to 1, measures a model's ability to distinguish between positive and negative classes, with values closer to one indicating better discrimination [45]. The C‐index measures a model's discriminative ability to rank individuals from lowest to highest risk, with values nearing 1 indicating perfect prediction [46]. The Brier score evaluates both discrimination and calibration, with a lower score, typically less than 0.25, indicating greater model accuracy [46]. Additionally, we conducted a Decision Curve Analysis (DCA), a method for evaluating the clinical utility of our developed models [47]. Clinical utility is depicted by the net benefit, ranging from negative infinity to a theoretical maximum, aligning with the incidence of the desired outcome for an impeccably accurate model (inherently less than 1.0).

### 2.8 Feature Importance

The importance of model inputs or features was determined using an explainable AI (XAI) method, the permutation‐based feature importance method. This model interpretation technique explores which inputs significantly affect the model prediction and is highly recommended in *black‐box* models. There are various ways to estimate feature importance from models [23], but the permutation‐based importance approach is a model‐agnostic method that is easy to understand and implement. Permutation feature importance measures the change in metric (e.g., C‐index) or model error after shuffling the values of a single input feature [48]. That is, the importance score for a feature is determined by the difference between the original error/metric and the permuted error/metric. Important features will result in a significant increase in error or decrease in metric when permuted, indicating the model's reliance on them for making predictions. Conversely, features that are not important will show minimal change in error/metric upon permutation, reflecting that the features are less important.

### 2.9 Statistical Analyses

Each analysis, including predicting CVD risk, is conducted separately for men and women. Statistical analyses were undertaken using Stata v.17 and R v.4.0.2. Continuous variables were expressed as means and standard deviations (SD) or medians and interquartile ranges (IQR) depending on their distribution. Categorical variables were characterized using frequencies and percentages. R was used for imputation using the *missRanger* package. The ML and DL models, including the CoxPH model, were conducted using the *Pysurvival* package in Python version 3.8.

### 2.10 Sensitivity Analysis

While integrating SDoH into CVD risk prediction models helps to get more accurate and equitable risk assessments, in this study, CVD risk was also predicted using the established CVD risk factors and the best fitted models.

## 3 Results

### 3.1 Basic Characteristics

The study included 12,896 Australian participants, comprising 5884 men (training data = 4118 and testing data = 1766) and 7012 women (training data = 4908 and testing data ==2, 104), all aged 70 years or older (Figure S1.). The median age of men was 73.8 years (IQR: 71.6–77.3) in the training data and 73.9 years (IQR: 71.7–77.5) in the testing data. For women, the median age was 74.1 years (IQR: 71.8–77.8) in both the training and testing datasets. During a median follow‐up period of 8.3 years (IQR: 7.2–9.5) for men, 476 individuals (11.6%) in the training and 231 individuals (13.1%) in the testing data developed CVD. For women, during a median follow‐up period of 8.5 years (IQR: 7.3–9.6), 404 women (8.2%) in the training data and 169 women (8.0%) in the testing data developed CVD. Furthermore, participant characteristics in both the training and testing datasets is summarized in Table S4.

### 3.2 Correlation Between Candidate Predictors and Feature Selection

#### 3.2.1 Correlation Between Features

The correlation matrix showing the correlation coefficient for men and women in the training dataset is presented in Figures S2 and S3, respectively. Except for partnership and living arrangement (with an absolute correlation coefficient of ~0.84), there were no significant correlations between the candidate predictors in both men and women.

#### 3.2.2 Feature Selection

**FIGURE 3:**
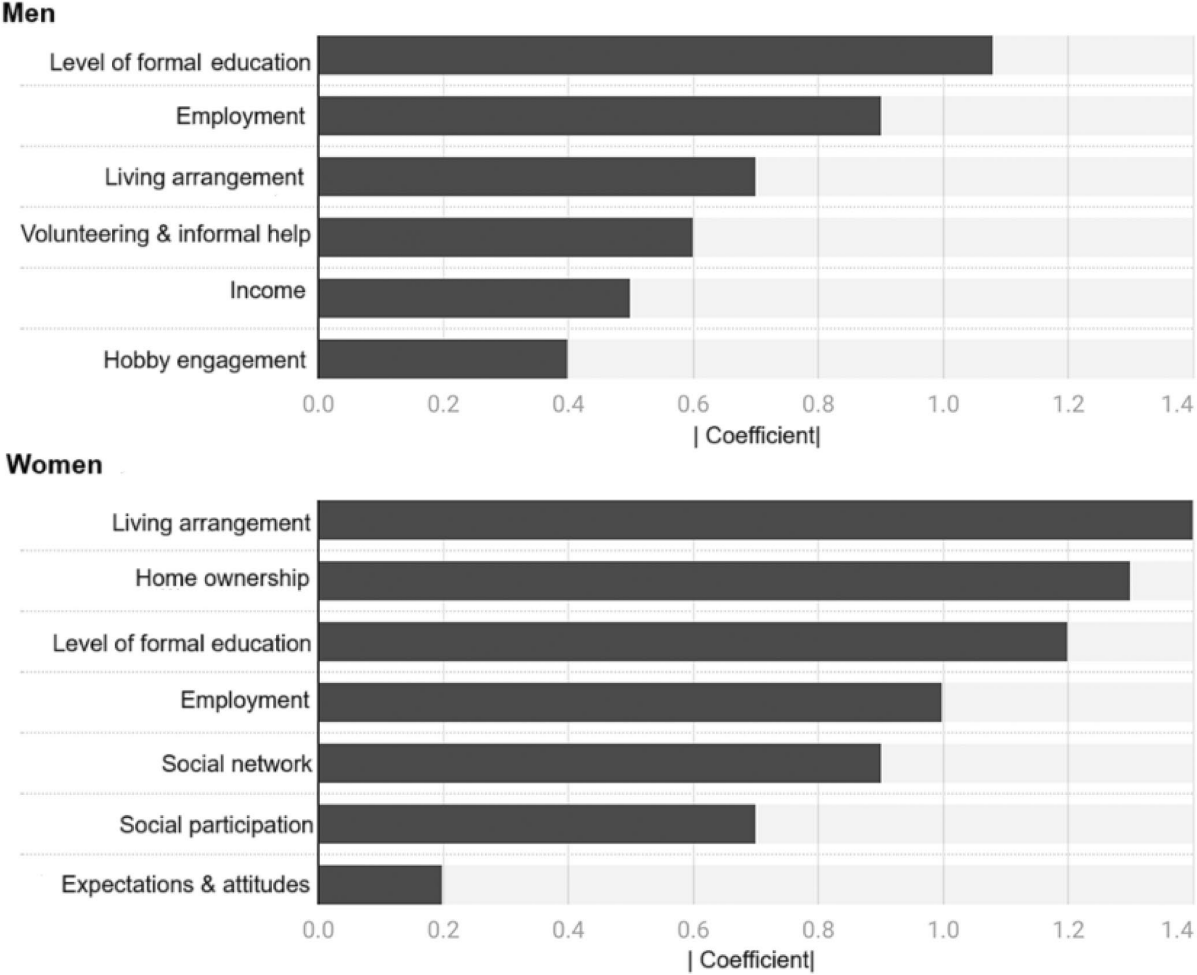
Social determinants of health variables selected using the Elastic Net method among men and women. The variables are presented from most important to least important based on the absolute values of their coefficients.

Of the correlated variables mentioned above, living arrangement was selected, as it can also encapsulate partnership status and offers a wider view of an individual's social context. Then, six SDoH variables for men and seven for women were selected using the Elastic Net method. The details of the selected SDoH variables are presented in Figure 3.

### 3.3 Model Performance Evaluation

The prediction performance evaluation results for the four models are detailed in Table 1. Based on the C‐index and AUC values, the RSF model (C‐index = 0.732; 5‐year AUC = 0.657; and 10‐year AUC = 0.676) among men and the DeepSurv model (C‐index = 0.670; 5‐year AUC = 0.676; and 10‐year AUC = 0.677, which is comparable to the 10‐year AUC of the RSF model) among women performed best. The IBS, a single summary measure of prediction error over time, of the models was also lower (less than 0.25), indicating low prediction error (Table 1 and Figure S4). Additionally, the DCA demonstrates the superior performance of the RSF, DeepSurv, and NMTLR models compared to the standard CoxPH model (Figure S5).

**TABLE 1:** Performance of the four survival models.

Models	Concordance index^a^ ^c^		Integrated brier score (IBS)^b^ ^d^	The area under the receiver operating characteristics curve (AUC)^b^ ^c^	
	Train	Test		5‐year	10‐year
Men					
CoxPH	0.570	0.597	0.074	0.578 (0.523, 0.632)	0.539 (0.491, 0.588)
RSF	0.720	0.732	0.071	0.657 (0.601, 0.713)	0.676 (0.631, 0.722)
DeepSurv	0.684	0.660	0.066	0.629 (0.575, 0.683)	0.660 (0.614, 0.706)
NMTLR	0.680	0.647	0.072	0.600 (0.543, 0.657)	0.659 (0.612, 0.705)
Women					
CoxPH	0.638	0.632	0.045	0.654 (0.597, 0.711)	0.648 (0.600, 0.695)
RSF	0.694	0.653	0.047	0.661 (0.596, 0.726)	0.679 (0.631, 0.728)
DeepSurv	0.690	0.670	0.042	0.676 (0.615, 0.738)	0.677 (0.630, 0.725)
NMTLR	0.686	0.664	0.048	0.671 (0.609, 0.734)	0.665 (0.616, 0.714)

Abbreviations: CoxPH, Cox proportional hazards model; NMTLR, neural multi‐task logistic regression; RSF, random survival forest.

^a^ Concordance index was calculated for training and testing data.

^b^ Integrated brier score (IBS) and the area under the receiver operating characteristics curve (AUC) were calculated based on the test dataset.

^c^ C‐index and AUC provide information about a model's discriminative ability. Both metrics range from 0 to 1.0. A C‐index or AUC of 0.5 indicates no discrimination (random guessing), while a value approaching 1.0 indicates better discrimination.

^d^ The Brier score evaluates both discrimination and calibration, with a lower score, typically less than 0.25, indicating greater model accuracy.

### 3.4 Feature Importance

**FIGURE 4:**
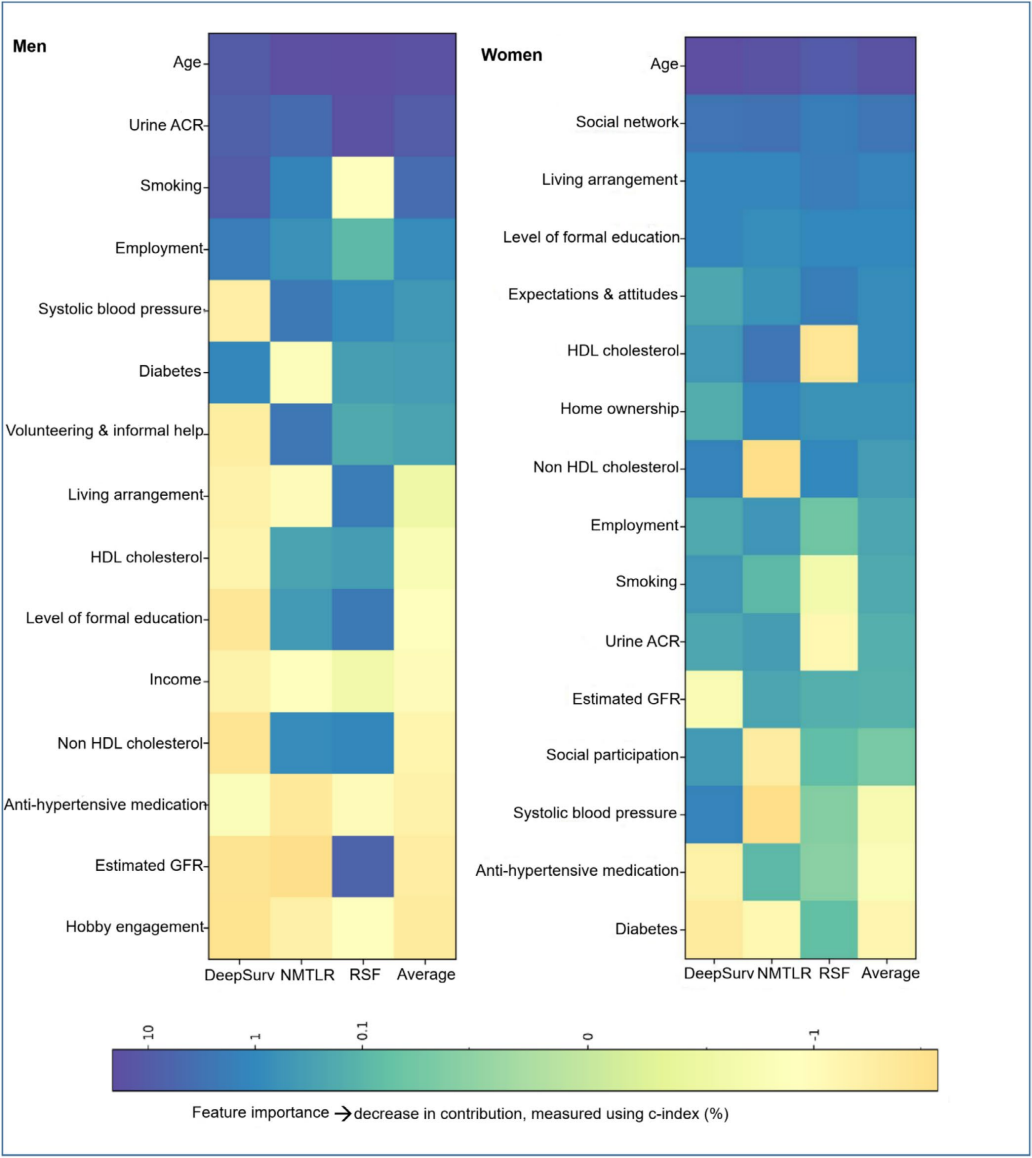
Heatmap showing permutation‐based feature importance for men and women. The values indicate a decrease in C‐index when a single feature value is randomly shuffled, with higher values indicating the feature is more important in influencing the predictive accuracy of the model. If the reduction is less than 0%, the feature is deemed uninformative. ACR, albumin‐to‐creatinine ratio; GFR, glomerular filtration rate; HDL, high density lipoprotein; NMTLR, neural multi‐task logistic regression; RSF, random survival forest.

Among men, age, urine ACR, and smoking were the top three predictors of incident CVD, while SDoH variables, such as employment, volunteering, living arrangement, and education, were among the top 10 predictors of incident CVD. For women, age was the top predictor of incident CVD, followed by SDoH variables, such as social network, living arrangement, education, and expectations and attitudes (Figure 4).

### 3.5 Sensitivity Analysis

When the traditional CVD risk factors were considered, the performance of the RSF for men was as follows: C‐index = 0.724; IBS = 0.071, the 5‐year AUC = 0.656 (95% concordance index (CI): 0.60, 0.712); and the10‐year AUC = 0.678 (95% CI: 0.632, 0.723). For women, the DeepSurv model had a C‐index of 0.646, IBS of 0.043, and the 5‐year and 10‐year AUC of 0.661 (95% CI: 0.597, 0.724) and 0.650 (95% CI: 0.600, 0.700), respectively.

## 4 Discussion

### 4.1 Main Findings

This study developed gender‐specific CVD risk prediction models by integrating SDoH and the established CVD risk factors. RSF for men and DeepSurv for women had better performance in predicting CVD risk. SDoH have appeared as important predictors of CVD risk, especially for women. In brief, among men, the strongest predictors were age, urine ACR, and smoking. SDoH variables such as employment, volunteering, living arrangement, and education were also among the top ten predictors. While for women, following age, the primary predictors were SDoH variables such as social network, living arrangement, education, and expectations and attitudes.

### 4.2 Comparison With Previous Works

Previous risk prediction models have not placed significant emphasis on SDoH. Only a handful of studies have incorporated SDoH into CVD prediction models. For example, a study done among breast cancer patients found that the inclusion of SDoH during predicting the 2‐year major adverse cardiovascular event (MACE) revealed an enhanced predictive accuracy, particularly among non‐Hispanic Black women [49]. A retrospective study utilized data from the Get With The Guidelines–Heart Failure (GWTG‐HF) registry, which included patients hospitalized with acute decompensated heart failure, and evaluated whether incorporating SDoH enhances the prediction of in‐hospital mortality [50]. The study suggested that ML models that incorporate SDoH could enhance the prediction of in‐hospital mortality following hospitalization, especially among Black adults. A further study involving African American adults indicated that incorporating SDoH did not enhance the overall predictive accuracy of the risk prediction model [51]. Similarly, a study aimed at assessing the impact of SDoH on health prediction in the Intensive Care Unit found that community‐level SDoH features might not enhance model performance [52].

The above studies were done among individuals with a certain health condition and among young adults. Additionally, they also did not assess the contribution of individual SDoH variables, except for one study where SDoH variables were identified as the most significant (top ranked) [51], surpassing even the established behavioral and biological risk factors. However, our study included relatively healthy older participants aged 70 and above at recruitment. In this study, we also interpreted our models to reveal the contribution of individual features to the prediction accuracy, particularly SDoH variables. Overall, we found an improvement in the overall prediction performance by incorporating SDoH variables, and SDoH variables were identified as the main predictors, particularly among women.

### 4.3 Gender Difference in CVD Risk Prediction

We identified gender differences in terms of overall prediction performance as well as predictive factors. RSF among men and the DeepSurv model among women were the best‐performing models. For men, SDoH were among the top ten predictors of CVD risk. Among women, SDoH variables emerged as the main predictors of CVD, second only to age. In brief, various SDoH factors can significantly predict CVD for both men and women, with a more pronounced impact for women. The sensitivity analysis also indicated that integrating SDoH into CVD risk prediction models for women could enhance the overall model accuracy. Additional evidence suggests that adverse SDoH such as limited employment opportunities, lower educational levels, and inadequate social support disproportionately impact women's health, with these effects intensifying with age [53, 54]. This is because women are likely to face unique factors that amplify the impact of SDoH on CVD risk. These are hormonal changes throughout their life, pregnancy‐related complications, caregiving stress, and work‐life balance challenges, higher prevalence of autoimmune disorders [55], and intimate partner violence. Our study highlights the need to strengthen gender‐disaggregated CVD risk assessment and interventions when considering SDoH, rather than a gender‐neutral approach.

### 4.4 Implications for Clinical Practice and Recommendations

The aim of this study was to evaluate the overall predictive performance of the AI‐based predictive models with the inclusion of SDoH and to comprehend the role of individual predictors to the model's predictive accuracy. In sum, this study finding implies that: (1) using ML and DL models instead of the standard CoxPH model predicts CVD risk better for men and women, (2) incorporating both SDoH and the traditional risk factors while predicting CVD provides a more comprehensive assessment than considering either alone, (3) while traditional CVD risk factors address the immediate physiological and behavioral contributors to disease, SDoH encompass broader social and environmental influences that can exacerbate these risks, and (4) comparable findings may be obtained by considering only the traditional risk factors of CVD; however, this approach may introduce biases and not fully capture the complex relationships between the traditional and SDoH variables. In conclusion, this study corroborates earlier studies advocating for multisectoral strategies to address adverse SDoH to enhance cardiovascular health [15, 56, 57]. One of such strategies is social prescribing (SP) [56, 58], which targets adverse SDoH such as poverty, unemployment, inadequate housing, and social isolation. Integrating SP into standard healthcare practices can effectively address these underlying causes of health disparities, enhance patient outcomes, and diminish healthcare expenditures.

This study, while informative, has its own limitations, leading to the following recommendations. Firstly, the study's cohort, selected from a long‐term clinical trial, may not be representative due to a healthy cohort effect and the inclusion of individuals with elevated levels of social confidence, health interest, and generosity. Secondly, SDoH‐sensitive models could potentially worsen biases and discrimination already present in healthcare systems [59, 60]. Therefore, it is crucial to employ detailed considerations tailored to the context, intent, and use of the developed predictive model. Thirdly, some relevant SDoH variables were not included in this study, such as early childhood adverse events, quality of social networks, and environmental factors (e.g., such as food environment and walkability). Additionally, SDoH are intricate and vary according to context and setting, requiring standardized tools to measure them. Considering the context and settings while measuring SDoH can assist in developing effective strategies that accurately address their impact on health outcomes. Fourthly, SDoH variables are interconnected, making isolating their individual effects on CVD challenging. Therefore, identifying significant patterns and assessing potential causal relationships could be crucial for developing effective interventions. Lastly, 99% of the participants from ASPREE who also participated in ALSOP self‐identified as White. Therefore, our findings may not be generalizable for other ethnic or racial groups, particularly the diverse cultural and linguistic communities in Australia. Hence, replicating this study across diverse ethnic groups and younger cohorts is warranted to ensure its broader applicability.

## 5 Conclusion

The study findings underscore the importance of integrating SDoH variables into CVD risk prediction models. SDoH can enhance overall CVD risk prediction and are among the main predictors for CVD risk, particularly among women, surpassing even the well‐established CVD risk factors. Therefore, enhancing strategies to mitigate CVD risk beyond primary care is crucial. This involves comprehending the upstream factors that affect patient health and pinpointing those who require referral to community resources, such as SP programs.

## Author Contributions

Study concept: Achamyeleh Birhanu Teshale and Rosanne Freak‐Poli. Study design: Achamyeleh Birhanu Teshale, Htet Lin Htun, Mor Vered, and Rosanne Freak‐Poli. Acquisition of data: Achamyeleh Birhanu Teshale and Rosanne Freak‐Poli. Statistical analysis: Achamyeleh Birhanu Teshale, Mor Vered, and Rosanne Freak‐Poli. Interpretation of results: All authors. Drafting of the manuscript: Achamyeleh Birhanu Teshale. Critical revision of the manuscript for important intellectual content: All authors. Supervision: Alice J. Owen, Mor Vered, and Rosanne Freak‐Poli. All authors have approved the final manuscript.

## Disclosure

Funders played no role in the design of the study, data collection, analysis and interpretation of data, decision to publish, and in the writing of the manuscript.

## Ethics Statement

ASPREE trial and ALSOP sub‐study were conducted in accordance with the Declaration of Helsinki 1964 as revised in 2008. The ASPREE trial was approved by multiple Institutional Review Boards in Australia and the U.S. (www.aspree.org). ALSOP has been reviewed and approved by the Monash University Human Research Ethics Committee (Social ALSOP: CF11/1935–2,011,001,094). All participants in the ASPREE clinical trial and ALSOP sub‐study signed informed consent for participation. The present study has been approved by the Monash University Human Research Ethics Committee to conduct secondary data analysis (Project ID: 35233).

## Conflicts of Interest

The authors declare no conflicts of interest.

## Supporting information


**Table S1.** Variable measurement.
**Table S2.** Hyperparameters utilized for prediction.
**Table S3.** Number and proportion of missing data.
**Table S4.** Basic characteristics of study participants in the training and testing dataset.
**Figure S1.** Flow chart of sampling procedure and sample size.
**Figure S2.** Correlation plot for candidate variables in men.
**Figure S3.** Correlation plot for candidate variables in women.
**Figure S4.** Brier score or prediction error curve among (a) men and (b) women.
**Figure S5.** Comparison of models using Decision curve analysis in (a) men and (b) women.

## Data Availability

The ASPREE and ALSOP are not publicly available since they are ongoing. However, they are available to partnering and external researchers for projects of appropriate scientific merit, and expressions of interest to analyze data from these datasets is coordinated through the ASPREE Access Management Site (AMS) (https://aspree.org/aus/researchers/).
