# Supplementary material for: Integrating Social Determinants of Health and Established Risk Factors to Predict Cardiovascular Disease Risk Among Healthy Older Adults
Source: J Am Geriatr Soc. 2025 Mar 18;73(6):1797–807. doi: 10.1111/jgs.19440 (PMC12205292; doi:10.1111/jgs.19440)
Supplement: Supplementary file 1 — Table S1. Variable measurement. Table S2. Hyperparameters utilized for prediction. Table S3. Number and proportion of missing data. Table S4. Basic characteristics of study participants in the training and testing dataset. Figure S1. Flow chart of sampling procedure and sample size. Figure S2. Correlation plot for candidate variables in men. Figure S3. Correlation plot for candidate variables in women. Figure S4. Brier score or prediction error curve among (a) men and (b) women. Figure S5. Comparison of models using Decision curve analysis in (a) men and (b) women. [file JGS-73-1797-s001.pdf]

Integrating social determinants of health and established risk factors to predict cardiovascular disease risk among healthy older adults

Supplemental Material

Supplemental Tables

Table S1. Variable measurement.

Table S2. Hyperparameters utilised for prediction.

Table S3. Number and proportion of missing data

Table S4. Basic characteristics of study participants in the training and testing dataset

Supplemental figures

Figure S1. Flow chart of sampling procedure and sample size

Figure S2. Correlation plot for candidate variables in men.

Figure S3. Correlation plot for candidate variables in women.

Figure S4. Brier score or prediction error curve among (a) men and (b) women.

Figure S5. Comparison of models using Decision curve analysis in (a) men and (b) women.

Table S1. Variable measurement

Variables	Measurement	Remark
**Demographics**		
Gender	Men and women	
Age	Age in years measured as continuous variable	
Race	White/Caucasian and others (Aboriginal/TSI, Native Hawaiian/Other Pacific Islander/Maori, Asian, American Indian, Black/African American, more than one race, others)	Not included in the prediction model because of the small sample in the 'other' group
**Social Determinants of Health**		
**Economic Stability**		
Current employment status	Employed (Full/Part time) and not employed	
Household income (per year)	High income (≥$50,000, those preferred not to answer considered here) and Low (<$50,000)	
Homeownership	Defined as owned if either the participant or spouse/partner has a house and not otherwise	
**Education Access and Quality**		
Level of formal education	High (>12 years of education) and Low (≤12 years of education)	
First language	English and non-English	
**Social and Community Context**		
Living arrangement	A binary variable coded as living with others (family/spouse/friends) and living alone	
Partnership status	A binary variable coded as partnered (includes married and De Facto relationship) and not partnered	
Social network	Based on Lubben Social Network Scale-6 (LSNS-6). LSNS-6 consists of six questions, each scored from 0 to 5, concerning the number of relatives and friends (separately) a person contacts at least monthly (1).<br>The questions are:<br>1) How many relatives and friends do you see or hear from at least once a month?<br>2) How many relatives and friends do you feel comfortable discussing private matters with?<br>3) How many relatives and friends do you feel close enough to that you could ask them for help?<br>The total score is the sum of the six items, with five possible responses each, ranging from 0 to 30.	Original responses:<br>0) None<br>1) One<br>2) Two<br>3) Three or four<br>4) Five through eight<br>5) Nine or more

	An optimal cutoff point to differentiate high social engagement from low social engagement (social isolation) was determined as proposed by Lubben et al., where a score of 12 or lower indicates social isolation.	
Social participation/activity	Consistent with other research (2, 3), social participation was gauged using five specific items or questions.<br>How frequently do you:<br>1) Go to a club, local organisation, neighbourhood or other small group?<br>2) Go to church, temple or other places of worship, or take part in related activities?<br>3) Do you go to museums, galleries or exhibitions?<br>4) Do you go to an education class?<br>5) Do you go to the cinema, theater or other social/sporting entertainment?<br>In this study, social participation was categorised as adequate/high if individuals belonged to or participated in any of the aforementioned activities at least once per month, and as inadequate/low if they did not.	Original Responses:<br>1) Never<br>2) Less than once a month<br>3) 1-3 times a month<br>4) Once a week or more<br>5) Most days
Volunteering and informal help	Four variables were utilised to assess volunteering: providing care for other adults in the past year, offering primary care for sick children during adulthood, volunteering for babysitting in the past year, and engaging in unpaid volunteer work (excluding babysitting and caregiving). Volunteering was then classified as "Yes" if the participant engaged in any of the aforementioned activities, and "No" if they did not.	Original responses:<br>Volunteering primary care for sick children and currently doing unpaid volunteer work had binary responses (yes-no type).<br>Babysitting and care provider for adults during the past year was measured using 0-3 Likert Scales.<br>0) Never<br>1) Occasionally (once a week)<br>2) Often (more than once a week)<br>3) Every/most days<br>These two variables were recoded into yes (participated for at least

		once a week) and no (never participated).
Hobby engagement	A composite variable was assessed using nine activities: listening to the radio or music, reading books, newspapers, or magazines, playing games, engaging in puzzles or crosswords, crafting, writing letters, using a computer, cooking, and painting or drawing. Initially, each activity was recoded from five possible responses to a binary scale: at least once a month (Never and Rarely; coded as "0") and less than once a month (coded as "1"). Subsequently, the composite score (ranges from 0-9) was treated as a continuous variable.	
Expectations and attitudes (optimism–pessimism)	Measured based on the six items of the Life Orientation Test Revised (LOT-R) (4):<br>1) In uncertain times, I usually expect the best ®.<br>2) If something can go wrong for me, it will.<br>3) I'm always optimistic about my future ®.<br>4) I hardly ever expect things to go my way.<br>5) I rarely count on good things happening to me.<br>6) Overall, I expect more good things to happen to me than bad ®.<br>Each item is rated on a 5-point scale. A composite score is then created, ranging from 0-24, with higher scores indicating greater levels of optimism.	Original responses:<br>0) Strongly agree.<br>1) Agree a little<br>2) Neither agree nor disagree<br>3) Disagree a little<br>4) Strongly disagree<br><br>® Reverse coded
Difficulties and stressful life events	Ten stressful life events from the past year were assessed with 'yes' or 'no' responses. These included: 1) death of a spouse/partner, 2) serious illness in a spouse/partner, 3) death or serious illness in family members or close friends, 4) major financial problems, 5) divorce or breakup with a spouse or partner, 6) divorce or breakup in family members or close friends, 7) major conflict with children or grandchildren, 8) major accidents, disasters, muggings, unwanted sexual experiences, robberies, or similar events, 9) job loss or retirement in family members or close friends, and 10) death of a pet. Following previous literature (5), we computed a continuous stressful life events variable by summing the number of events and categorised the score accordingly into three categories (number of past-year adverse events: 0 event, 1 event, 2 event, and 3 or more events).	
Depressive symptoms	The Center for Epidemiologic Studies Depression Scale (CESD-10) was utilised in this study (6). Then a score of 8 or higher was used to indicate the presence of depression.	
**Neighbourhood and built environment**		
Remoteness	Participants' rurality is categorised into three areas: Major cities, Inner regions, and Outer regions of Australia.	
Socio-Economic Indexes for Areas (SEIFA)	The Socio-Economic Indexes for Areas-Index of Relative Socioeconomic Advantage and Disadvantage (SEIFA-IRSAD) was determined using the residential postcodes of participants, drawing on data from the 2011 Australian Census. The calculation of the	

	SEIFA-IRSAD index takes into account factors such as annual household income, education, occupation, internet access, and the rental rates of dwellings in a given area. The study took into account the SEIFA-IRSAD scores, which were calculated in quintiles.	
Transport (satisfaction)	Transport satisfaction was assessed with a single item on a 5-point scale. In this study, responses were recoded as "Satisfied" if participants were very satisfied or moderately satisfied, and as "Not Satisfied" if they were very dissatisfied, somewhat dissatisfied, or neither.	Original responses:<br>1) Very satisfied<br>2) Moderately satisfied<br>3) Neither satisfied nor dissatisfied<br>4) Somewhat dissatisfied<br>5) Very dissatisfied
**Healthcare quality and access**		
Health insurance	Health care insurance is categorized as a binary variable, coded as either insured or uninsured	
**Standard modifiable risk factors**		
Systolic blood pressure	The mean value from three measurements in a sitting position was used as a continuous variable	
Taking antihypertensive medications	The prescription of antihypertensive agents was coded by using the Anatomical Therapeutic Chemical codes (7) and included the categories C02 (antihypertensives), C03 (diuretics), C07 (beta blocking agents), C08 (calcium channel blockers), and C09 (agents acting on the renin-angiotensin system)	
Urine albumin to creatinine ration (ACR)	The urine albumin to creatinine ratio, expressed in mg/mmol, is utilised as a continuous variable	
Estimated glomerular filtration rate (GFR)	Estimated glomerular filtration rate in ml/min/m^2 as a continuous variable	
Current smoking	Whether the participants were smokers or not at enrollment.	
HDL cholesterol	High-density lipoprotein cholesterol is measured in mmol/L and utilised as a continuous variable	
Non-HDL cholesterol	Non-high-density lipoprotein cholesterol (all cholesterol other than HDL) is measured in mmol/L and is regarded as a continuous variable	
Diabetes	The presence of diabetes was based on participants' report of diabetes mellitus or a fasting glucose level of at least 126 mg per deciliter (≥7 mmol per liter) or receipt of treatment for diabetes (8).	

Table S2. Number and proportion of missing values.

Characteristics	Men	Women	Remark
	Number missing (%)	Number missing (%)	
Age in years	0 (0.0)	0 (0.0)	
Race	6 (0.1)	1 (0.01)	
Level of education	0 (0.0)	0 (0.0)	
Household income per year	212 (3.6)	485 (6.9)	
Current employment	168 (2.9)	209 (3.0)	
Home ownership	105 (1.8)	174 (2.5)	
Living arrangement	0 (0)	0 (0.0)	
Partnership status	0 (0.0)	0 (0.0)	
Volunteering babysitting last year	142 (2.4)	232 (3.3)	Used to measure volunteering or informally helping others
Volunteering primary carer last year	153 (2.6)	309 (4.4)	
Volunteering primary carer for children in the past	174 (3.0)	287 (4.1)	
Volunteering unpaid volunteer currently (not including babysitting or child minding)	98 (1.7)	167 (2.4)	
How often do you:			
Go to a club, local organisation, neighbourhood or other small group?	117 (2.0)	230 (3.3)	Used to measure social participation
Go to church, temple or other places of worship, or take part in related activities?	146 (2.5)	251 (3.6)	
Do you go to museums, galleries or exhibitions?	163 (2.8)	285 (4.1)	
Do you go to an education class?	298 (5.1)	530 (7.6)	
Do you go to the cinema, theater or other social/sporting entertainment?	106 (1.8)	156 (2.2)	
How often do you do the following?			
Listen to radio or music	131 (2.2)	171 (2.4)	Used to measure hobby engagement
Read books, newspapers or magazine	122 (2.1)	143 (2.0)	
Play games such as cards or chess	149 (2.5)	252 (3.6)	
Do puzzles or crosswords	138 (2.3)	202 (2.9)	
Skew, knit, do woodwork, metalwork, tinkering or other craftwork	119 (2.0)	165 (2.4)	
Use a computer (including an internet)	105 (1.8)	177 (2.5)	
Compose a letter, journal or other written work (typed or hand-written)	93 (1.6)	169 (2.4)	
Paint or draw	131 (2.2)	252 (3.6)	

Cook	87 (1.5)	108 (1.5)	
How many relatives (including spouses, partners, children, etc.) do you see or hear from at least once a month?	42 (0.7)	64 (0.9)	Used to measure social network
How many relatives do you feel at ease with, that you can talk to about private matters?	66 (1.1)	85 (1.2)	
How many relatives do you feel close to, such that you could call on them for help?	52 (0.9)	76 (1.1)	
How many friends do you see or hear from at least once a month?	53 (0.9)	61 (0.9)	
How many friends do you feel at ease with, that you can talk to about private matters?	62 (1.1)	79 (1.1)	
How many friends do you feel close to, such that you could call on them for help?	56 (1.0)	72 (1.0)	
Over the past year:			
Did your spouse or partner die	95 (1.6)	238 (3.4)	Used to measure stressful life events
Did you spouse or partner have a serious illness	216 (3.7)	529 (7.5)	
Did a close friend or family member (other than spouse or partner) die or have a serious illness	109 (1.9)	249 (3.6)	
Did you have any major problem with money	93 (1.6)	178 (2.5)	
Did you have a divorce or break up with a spouse or partner	99 (1.7)	216 (3.1)	
Did a close friend or family member have a divorce or break up	87 (1.5)	142 (2.0)	
Did you have a major conflict with children or grandchildren	83 (1.4)	140 (2.0)	
Did you have any major accidents, disasters, muggings, unwanted sexual experiences, robberies or similar events	67 (1.1)	128 (1.8)	
Did a family member or close friendship lose their job or retire	71 (1.2)	142 (2.0)	
Did a pet die	111 (1.9)	187 (2.7)	
In uncertain time, I usually expect the best	178 (3.0)	384 (5.5)	Expectations and attitudes (based on Life Orientation Test-Revised (LOT-R)
If something can go wrong for me, it will	222 (3.8)	404 (5.8)	
I am always optimistic about the future	163 (2.8)	322 (4.6)	
I hardly ever expect things to go my way	191 (3.2)	370 (5.3)	
I rarely count on good things happening to me	186 (3.2)	363 (5.2)	
Overall, I expect more good things to happen to me than bad	113 (1.9)	200 (2.9)	
Depressive symptoms	0 (0.0)	0 (0.0)	
Health insurance	0 (0.0)	0 (0.0)	
How satisfied are you with your ability to get to and from the places you want to go?	75 (1.3)	109 (1.6)	Used to measure satisfaction on transportation

First language	0 (0.0)	0 (0.0)	
Remoteness	10 (0.2)	22 (0.3)	
Socio-Economic Indexes for Areas (SEIFA)	10 (0.2)	22 (0.3)	
Smoking history	0 (0.0)	0 (0.0)	
HDL cholesterol in mmol/L	162 (2.8)	172 (2.5)	Used to measure non-HDL cholesterol<br>Mean (SD) (men: 3.6 (0.9); and women: 3.7 (1.0))
Total cholesterol in mmol/L	66 (1.1)	71 (1.0)	
Diabetes	0 (0.0)	0 (0.0)	
Antihypertensive medication use	0 (0.0)	0 (0.0)	
Systolic blood pressure in mm Hg	0 (0.0)	0 (0.0)	
Urine ACR in mg/mmol	315 (5.4)	377 (5.4)	
Estimated GFR in ml/min/1.73m^2	179 (3.0)	187 (2.7)	

Table S3. Hyperparameters utilised for prediction.

Models	Hyperparameters	
	Men	Women
Random Survival Forest (RSF)	Number of trees = 200, max features = sqrt, importance mode = normalized permutation, maximum depth = 5, minimum node size = 10, percentage of original samples used in each tree building = 0.63.	Number of trees = 200, max features = sqrt, importance mode = impurity, maximum depth = 5, minimum node size =20, percentage of original samples used in each tree building = 0.63.
DeepSurv	Number of layers = 1 [Layer(1): activation = ReLU, num_units = 150], Optimizer = adamax, lr = 3e-05, num_epochs = 4555, dropout = 0.2, l2_reg = 0.0026, batch_normalization = False.	Number of layers = 5 [Layer(1): activation = Sigmoid, num_units = 84; Layer(2): activation = CosReLU, num_units = 77; Layer(3): activation = LeCunTanh, num_units = 23; Layer(4): activation = Guassian, num_units = 9; Layer(5): activation = Tanh, num_units = 25], Optimizer = adam, lr = 0.00021, num_epochs = 3317, dropout = 0.3, l2_reg = 0.0031, batch_normalization = False.
Neural Multi-Task Logistic Regression (NMTLR)	Number of layers = 4 [Layer(1): activation = LogSigmoid, num_units = 66; Layer(2): activation = CosReLU, num_units = 84; Layer(3): activation = Swish, num_units = 98; Layer(4): activation = LeakyReLU, num_units = 68], Optimizer = rmsprop, lr = 0.0001, num_epochs = 1000, dropout = 0.2, l2_reg = 0.01, l2_smooth = 0.01, batch_normalization = False.	Number of layers = 4 [Layer(1): activation = Tanh, num_units = 25; Layer(2): activation = Hardtanh, num_units = 40; Layer(3): activation = ReLU, num_units = 83; Layer(4): activation = LeCunTanh, num_units = 27], Optimizer = rmsprop, lr = 0.00094, num_epochs = 444, dropout = 0.3, l2_reg = 0.0022, l2_smooth = 0.0086, batch_normalization = False.

Table S4. Basic characteristics of study participants in the training and testing dataset

Characteristic	Men (n = 5, 884[a])		Women (n= 7,012[a])	
	Training (n=4,118)	Testing (n=1,766)	Training (n=4,908)	Testing (n=2,104)
Age in years [Median (IQR)]	73.8 (71.6-77.3)	73.9 (71.7-77.5)	74.1 (71.8-77.8)	74.1 (71.8-77.8)
Level (years) of education (%)				
≤12 years	2,289 (55.6)	981 (55.6)	3,042 (62.0)	1,309 (62.2)
>12 years	1,829 (44.4)	785 (44.4)	1,866 (38.0)	795 (37.8)
Household income (%)				
Low	2,631 (64.0)	1,145 (64.8)	3,492 (71.1)	1,480 (70.3)
High	1,487 (36.0)	621 (35.2)	1,416 (28.9)	624 (29.7)
Current employment (%)				
Not employed	3,555 (86.3)	1,559 (88.3)	4,595 (93.6)	1,983 (94.3)
Full/part-time	563 (13.8)	207 (11.7)	313 (6.4)	121 (5.7)
Home ownership (%)				
Others	398 (10.0)	176 (10.0)	635 (12.9)	255 (12.1)
Self/spouse/partner	3,720 (90.3)	1,590 (92.0)	4,273 (87.1)	1,849 (87.9)
Living arrangement (%)				
Alone	686 (16.7)	292 (16.5)	1,987 (40.5)	878 (41.7)
With others (e.g., family, friends, and relatives)	3,432 (83.3)	1.474 (83.5)	2,921 (59.5)	1,226 (58.3)
Volunteering or informally helping others (%)				
No	988 (24.0)	430 (24.4)	476 (9.7)	212 (10.1)
Yes	3,130 (76.0)	1,336 (75.6)	4,432 (90.3)	1,892 (89.9)
Social participation (%)				
No	734 (17.8)	317 (18.0)	523 (10.7)	224 (10.7)
Yes	3,384 (82.2)	1,449 (82.0)	4,385 (89.3)	1,880 (89.3)
Hobby engagement [Mean (SD)]	5.1 (1.5)	5.0 (1.5)	6.0 (1.4)	6.0 (1.4)
Social network				
Low	353 (8.6)	164 (9.3)	325 (6.6)	149 (7.1)
High	3,765 (91.4)	1,602 (90.7)	4,583 (93.4)	1,955 (92.9)
Stressful life events (%)				
None	1,429 (34.7)	563 (32.0)	1,427 (29.1)	626 (29.7)
One	1,483 (36.0)	681 (38.5)	1,807 (36.8)	764 (36.3)
Two	786 (19.1)	330 (18.7)	1,004 (21.3)	435 (20.7)
Three or more	420 (10.2)	192 (10.8)	630 (12.8)	279 (13.3)
Expectations and attitudes [Median (IQR)]	17 (14-21)	17 (14-21)	18 (14-22)	18 (14-22)

Depressive symptoms (%)				
No	3,839 (93.2)	1,611 (91.2)	4,402 (89.7)	1,902 (90.4)
Yes	279 (6.8)	155 (8.8)	506 (10.3)	202 (9.6)
Health insurance (%)				
No	184 (4.5)	88 (5.0)	234 (95.2)	98 (4.7)
Yes	3,934 (95.5)	1,678 (95.0)	4,674 (95.2)	2,006 (95.3)
Transport satisfaction (%)				
Not satisfied	86 (2.1)	35 (2.0)	138 (2.8)	56 (2.7)
Satisfied	4,032 (96.9)	1,709 (96.8)	4,770 (97.2)	2,048 (97.3)
First language (%)				
Non-English	174 (4.2)	67 (3.8)	139 (2.8)	54 (2.6)
English	3,944 (95.8)	1,699 (96.2)	4,769 (97.2)	2,050 (97.4)
Remoteness (%)				
Major cities of Australia	1,463 (35.5)	635 (35.9)	1,734 (35.3)	744 (35.4)
Inner regional Australia	2,164 (52.6)	955 (54.1)	2,600 (53.0)	1,131 (53.7)
Outer regional Australia	491 (11.9)	176 (10.0)	574 (11.7)	229 (10.9)
SEIFA (%)				
Quintile 1	658 (16.0)	261 (14.8)	800 (16.3)	344 (16.4)
Quintile 2	698 (17.0)	304 (17.2)	810 (16.5)	367 (17.4)
Quintile 3	718 (17.4)	355 (20.1)	933 (19.0)	393 (18.7)
Quintile 4	781 (19.0)	332 (18.8)	963 (19.6)	404 (19.2)
Quintile 5	1,263 (30.6)	514 (29.1)	1,402 (28.6)	596 (28.3)
Current smoking (%)				
No	3,987 (96.8)	1,698 (96.1)	4,805 (97.9)	2,046 (97.2)
Yes	131 (3.2)	68 (3.9)	103 (2.1)	58 (2.76)
HDL cholesterol [Mean (SD)] in mmol/L	1.4 (0.4)	1.4 (0.4)	1.7 (0.5)	1.7 (0.5)
Non-HDL cholesterol [Mean (SD)] in mmol/L	3.6 (0.9)	3.6 (0.9)	3.7 (1.0)	3.7 (0.9)
Diabetes				
No	3,624 (88.0)	1,573 (89.1)	4,527 (92.2)	1,940 (92.2)
Yes	494 (12.0)	193 (10.9)	381 (7.8)	164 (7.8)
Antihypertensive medication use				
No	2,127 (51.7)	914 (51.8)	2,225 (45.3)	960 (45.6)
Yes	1,991 (48.3)	852 (48.2)	2,683 (54.7)	1,144 (54.4)
Systolic blood pressure [Median (IQR)] in mm Hg	141 (130-153)	142 (130-152)	137 (126-149)	137 (127-150)
Urine ACR [Median (IQR)] in mg/mmol	0.7 (0.4-1.4)	0.6 (0.4-1.2)	0.9 (0.5-1.5)	0.9 (0.5-1.4)
Estimated GFR ml/min/m^2 [Median (IQR)]	73.7 (64.4-84.0)	73.8 (64.1-82.8)	74.1 (63.4-84.3)	73.5 (63.2-84.2)

Survival years [Median (IQR)]	8.3 (7.2-9.5)	8.3 (7.2-9.5)	8.5 (7.3-9.6)	8.5 (7.4-9.7)
CVD[b] (%)				
No	3,642 (88.4)	1,535 (86.9)	4,504 (91.8)	1,935 (92.0)
Yes	476 (11.6)	231 (13.1)	404 (8.2)	169 (8.0)

[a]Missing data imputed using *missRanger* package in R for the training and testing data separately.

[b]Cardiovascular disease is measured by considering fatal coronary heart disease, hospitalisation due to heart failure, stroke, and myocardial infarction. Among men, in the training data, myocardial infarction occurred in 4.8% (n =196) of cases, hospitalisation due to heart failure in 2.3% (n = 95), stroke in 4.5% (n = 185), and fatal coronary heart disease in 3.0% (n = 124). Among men, in the testing data, myocardial infarction occurred in 5.5% (n = 104) of cases, hospitalisation due to heart failure in 2.6% (n = 45), stroke in 4.6% (n = 81), and fatal coronary heart disease in 3.7% (n= 65). In women, in the training data, there were 2.4% (n = 119) cases of myocardial infarction, 1.7% (n = 85) hospitalisation due to heart failure, 3.7% (n = 183) stroke cases, and 2.1% (n = 102) cases of fatal coronary heart disease. In women, in the testing data, there were 2.2% (n = 47) cases of myocardial infarction, 1.6% (n = 34) hospitalisation due to heart failure, 3.7% (n = 78) stroke cases, and 1.7% (n = 36) cases of fatal coronary heart disease.

Abbreviations: ACR, albumin to creatinine ratio; CVD, cardiovascular disease; HDL, high density lipoprotein, IQR: interquartile range; SD, standard deviation; SEIFA, socio-economic indexes for areas.

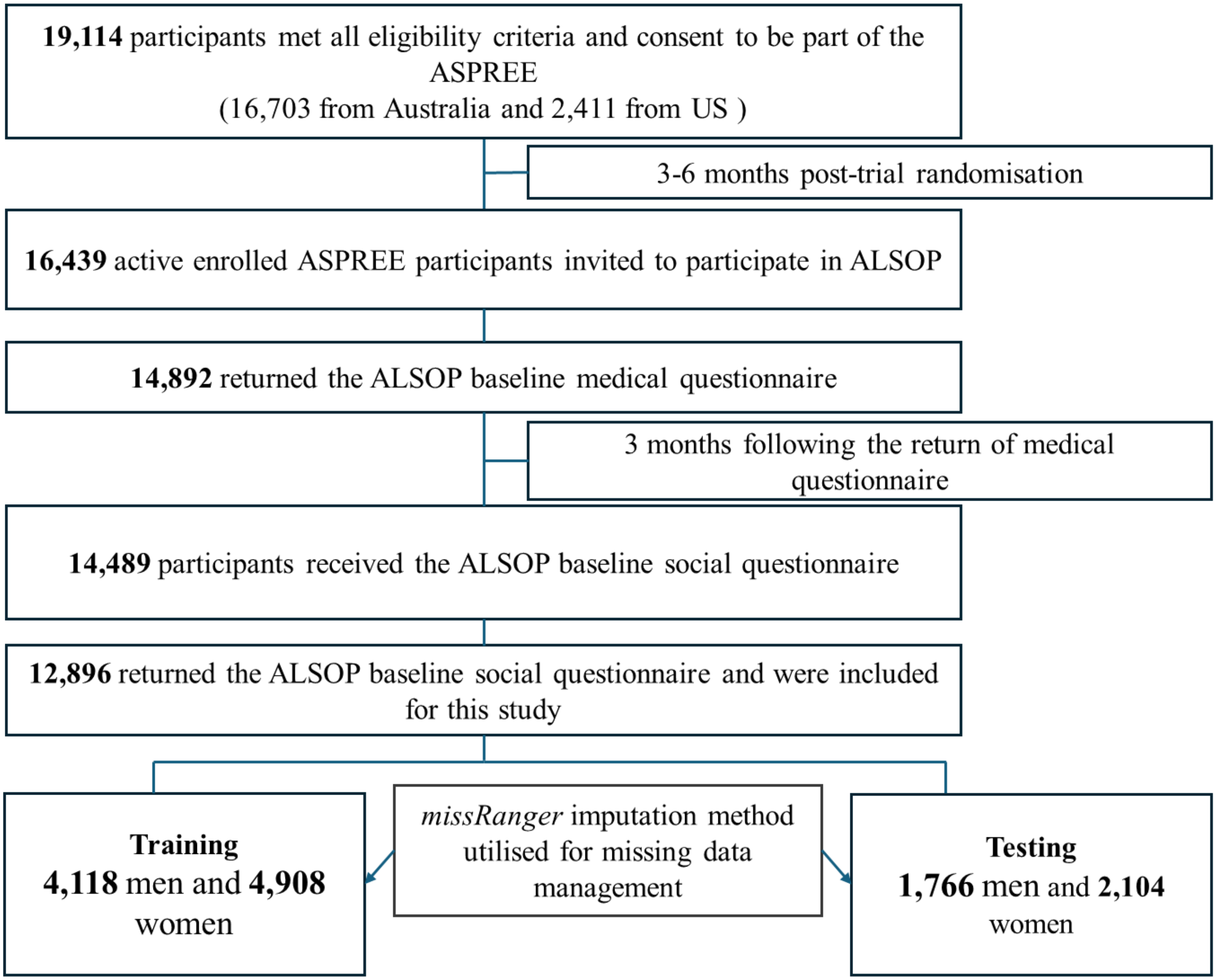

Figure S1. Flow chart of sampling procedure and sample size

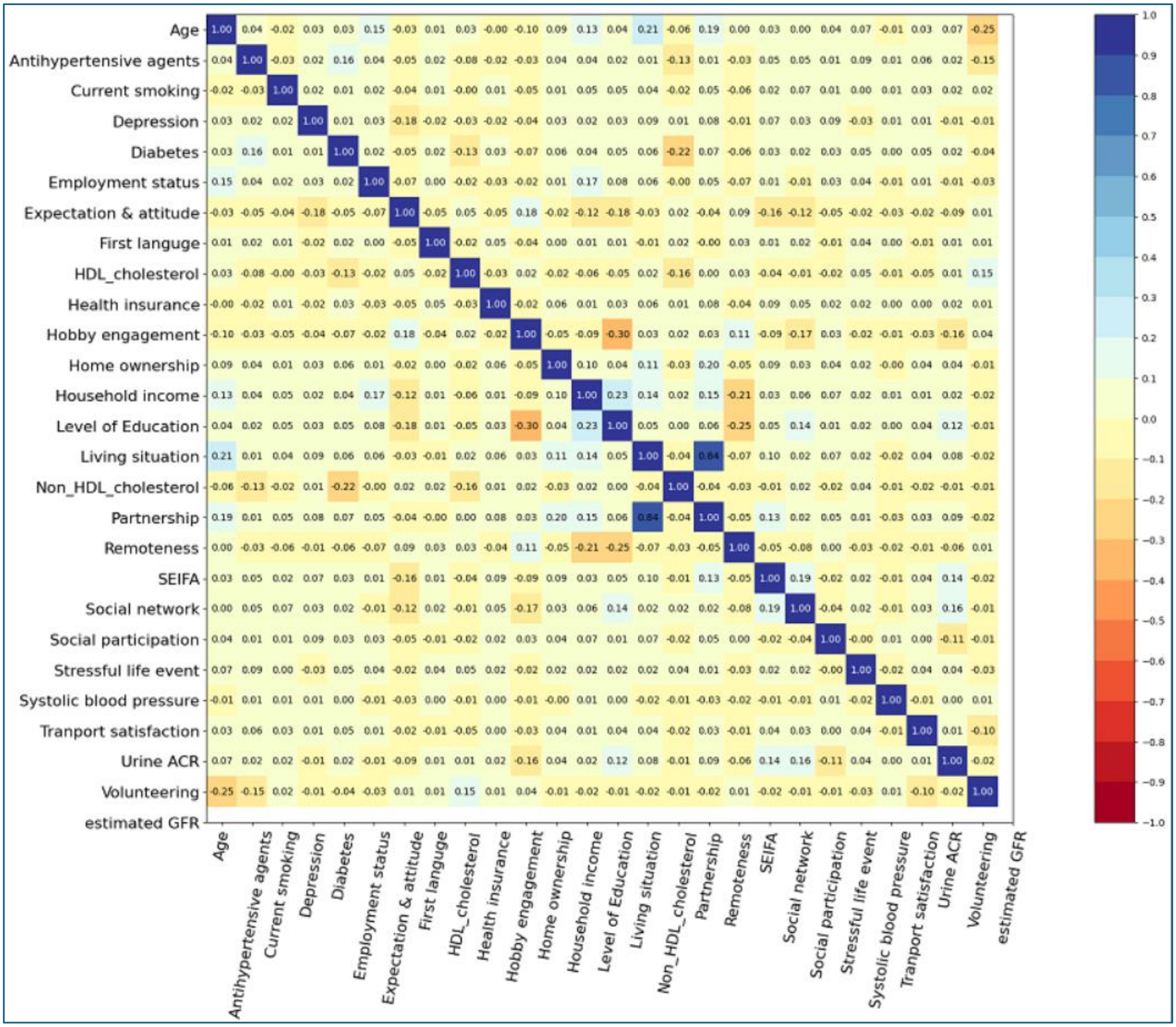

Figure S2. Correlation plot for candidate variables in men. Living situation (in this correlation plot) and living arrangement (throughout the manuscript) are similar terms. **Abbreviations:** ACR, albumin to creatinine ratio, GFR, glomerular filtration rate; HDL, high density lipoprotein; SEIFA, socio-economic indexes for areas

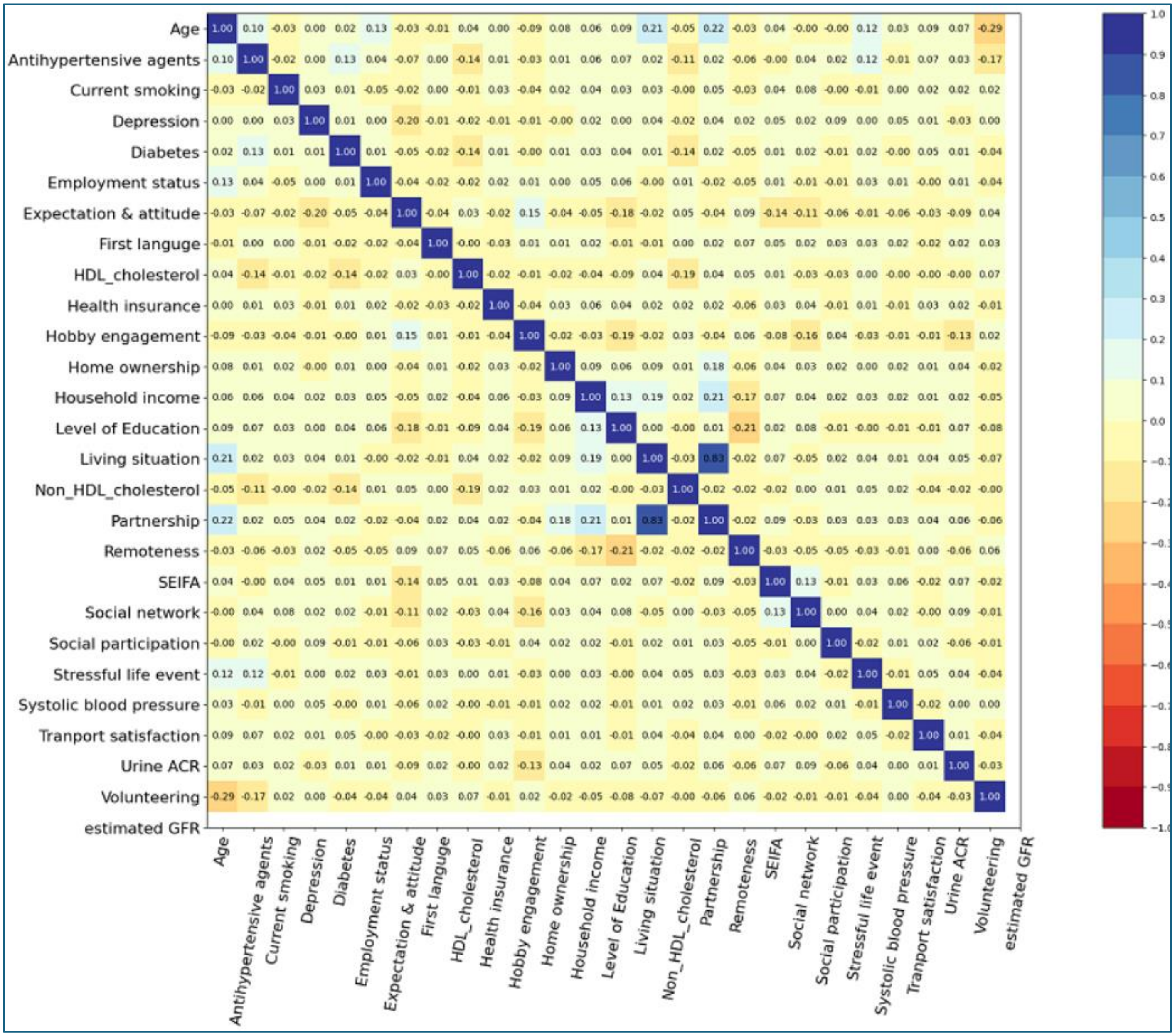

Figure S3. Correlation plot for candidate variables in women. Living situation (in this correlation plot) and living arrangement (throughout the manuscript) are similar terms. **Abbreviations:** ACR, albumin to creatinine ratio, GFR, glomerular filtration rate; HDL, high density lipoprotein; SEIFA, socio-economic indexes for areas.

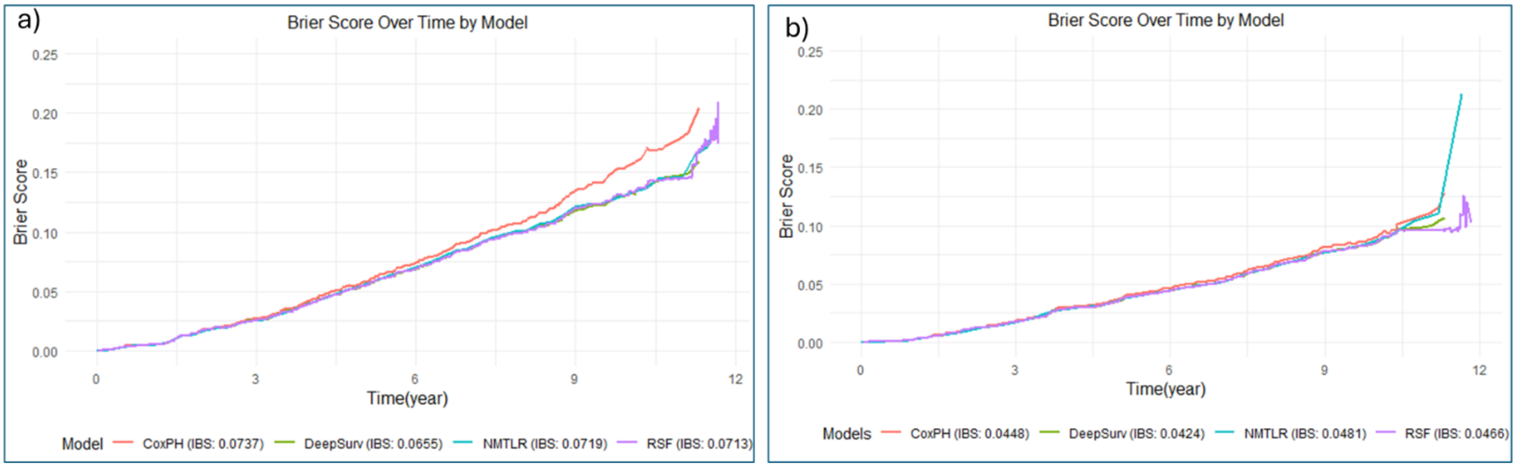

Figure S4. Brier score or prediction error curve among (a) men and (b) women. **Abbreviations:** CoxPH, Cox proportional hazards model; NMTLR, neural multi-task logistic regression; RSF, random survival forest.

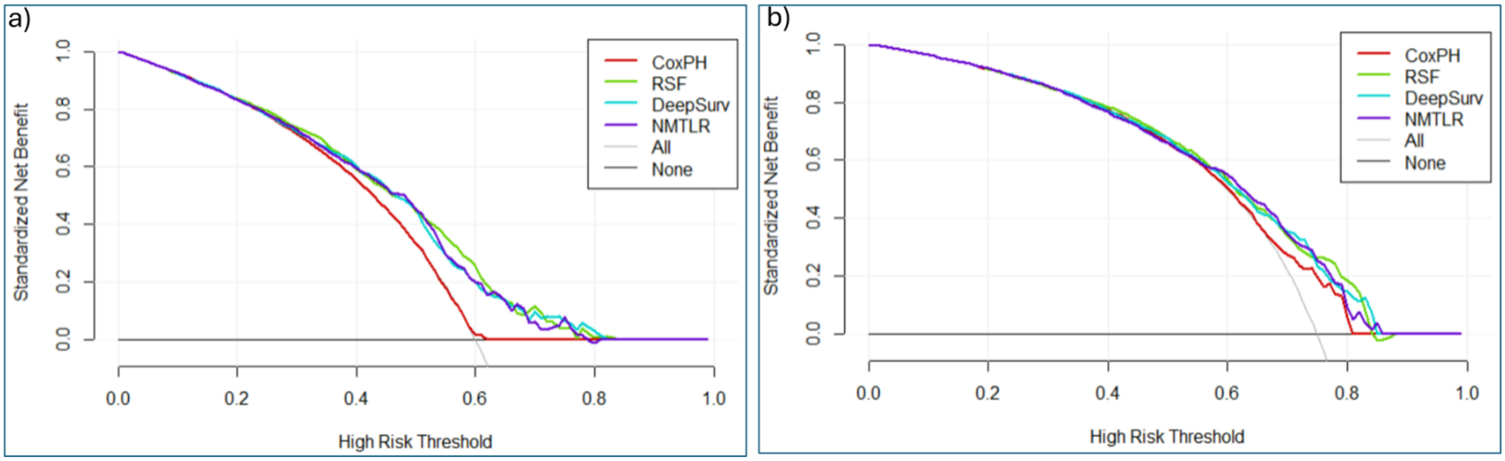

Figure S5. Comparison of models using Decision curve analysis in (a) men and (b) women. The y-axis measures the net benefit, which was calculated using true-positive and false-positive results. The machine learning and deep learning models had the highest net benefit at a threshold all positive (line labeled "All") and negative predictions (line labeled "None"), as well as the Cox model. **Abbreviations:** CoxPH, Cox proportional hazards model; NMTLR, neural multi-task logistic regression; RSF, random survival forest.

References

1. Lubben J, Blozik E, Gillmann G, Iliffe S, von Renteln Kruse W, Beck JC, et al. Performance of an Abbreviated Version of the Lubben Social Network Scale Among Three European Community-Dwelling Older Adult Populations. The Gerontologist. 2006;46(4):503-13.
2. Hwang J, Park S, Kim S. Effects of Participation in Social Activities on Cognitive Function Among Middle-Aged and Older Adults in Korea. Int J Environ Res Public Health. 2018;15(10).
3. Floud S, Balkwill A, Canoy D, Reeves GK, Green J, Beral V, et al. Social participation and coronary heart disease risk in a large prospective study of UK women. Eur J Prev Cardiol. 2016;23(9):995-1002.
4. Glaesmer H, Rief W, Martin A, Mewes R, Brähler E, Zenger M, et al. Psychometric properties and population-based norms of the Life Orientation Test Revised (LOT-R). British journal of health psychology. 2012;17(2):432-45.
5. Berntson J, Patel JS, Stewart JC. Number of recent stressful life events and incident cardiovascular disease: Moderation by lifetime depressive disorder. Journal of Psychosomatic Research. 2017;99:149-54.
6. Andresen EM, Malmgren JA, Carter WB, Patrick DL. Screening for depression in well older adults: Evaluation of a short form of the CES-D. American journal of preventive medicine. 1994;10(2):77-84.
7. Pratt NL, Kerr M, Barratt JD, Kemp-Casey A, Ellett LMK, Ramsay E, et al. The validity of the Rx-Risk comorbidity index using medicines mapped to the anatomical therapeutic chemical (ATC) classification system. BMJ open. 2018;8(4):e021122.
8. McNeil JJ, Wolfe R, Woods RL, Tonkin AM, Donnan GA, Nelson MR, et al. Effect of Aspirin on Cardiovascular Events and Bleeding in the Healthy Elderly. N Engl J Med. 2018;379(16):1509-18.
